# Primary mental healthcare for adults with mild intellectual disabilities: Patients’ perspectives

**DOI:** 10.1080/13814788.2024.2354414

**Published:** 2024-05-17

**Authors:** Katrien PM. Pouls, Mathilde Mastebroek, Suzanne J. Ligthart, Willem JJ. Assendelft, Geraline L. Leusink, Monique CJ. Koks-Leensen

**Affiliations:** Department of Primary and Community Care, Radboud University Medical Center, Nijmegen, the Netherlands

**Keywords:** Mild intellectual disability, primary health care, mental health, qualitative research, organisation of care

## Abstract

**Background:**

People with mild intellectual disabilities (MID) experience more mental health (MH) problems than the general population but often do not receive appropriate primary MH care. Primary MH care is essential in integrative MH care and, therefore, demands high quality. To improve primary MH care for this patient group, account must be taken of the experiences of people with MID. So far, their perspectives have been largely absent from primary MH care research.

**Objectives:**

To explore patients’ experiences, needs, and suggestions for improvement regarding primary MH care for people with MID.

**Methods:**

Qualitative study among adults with MID who visited their GP with MH problems in the previous 12 months. Semi-structured interviews were conducted using a guide based on Person-Centred Primary Care Measures. Transcripts were analysed thematically.

**Results:**

The 11 interviews that we conducted revealed four themes. The first theme, cumulative vulnerability, describes the vulnerability – instigated by the MID and reinforced by MH problems – experienced on a GP visit. The other themes (needs regarding the GP, needs regarding the network, self-determination) arise from this vulnerability.

**Conclusion:**

People with both MID and MH problems are extra vulnerable in primary care but desire self-determination regarding their MH care trajectory. This requires investment in a good GP-patient relationship and the organisation of additional support to meet these patients’ needs, for which collaborative care with the patient, the patient’s network, and other (care) professionals is of utmost importance.

## KEYMESSAGES

People with mild intellectual disabilities (MID) feel more vulnerable visiting their GP with mental health (MH) problems than with somatic problems

Patients with MH problems report additional needs and expectations regarding their care and support network

GPs face challenges in coordinating care for patients with both MID and MH problems

## Introduction

As general practitioners (GPs) are usually the first point of encounter for people with mild intellectual disabilities (MID) who have mental health (MH) problems, they have an essential role in the detection, treatment, and follow-up of MH problems. Although the prevalence of MH problems in people with MID is concerningly high, the primary MH care provided to this patient group is often insufficient. To improve primary MH care for this patient group, it is essential to consider the experiences of people with MID themselves. This qualitative research ascertains their experiences, needs, and suggestions for improvement regarding primary MH care, and thus generates insights into opportunities to improve primary MH care for people with MID.

People with mild intellectual disabilities (MID) are at high risk for mental health (MH) problems but often do not receive appropriate MH care [1–5]. As a result, people with MID may develop MH problems of a more severe or chronic nature, putting a high burden on people’s lives [6].

MID is characterised by significant limitations in intellectual functioning and adaptive skills, with onset during childhood [7,8]. Despite no intelligence quotient (IQ) range being included in the current Diagnostic and Statistical Manual of Mental Disorders (DSM-5) definition of MID [8], the 50–70 IQ range is often adhered to internationally, representing a significant limitation in intellectual functioning. However, a broader definition of MID is used in some countries, including the Netherlands. This broader definition includes an IQ range from 50 to 85, with such limitations in adaptive skills that support is needed to function adequately in daily life [9]. For this article, we used this broad MID definition.

In countries with a primary care model, such as the UK and the Netherlands, general practitioners (GPs) are the first point of encounter for people with MH problems and have an important role in detection, treatment, and follow-up. Primary MH care is seen as a key element of integrative MH care, which involves task-sharing with MH services and other care providers [10]. In the Netherlands, it is estimated that approximately 6.4% of the Dutch population have MID according to the broad MID definition [11], of whom up to a third experience MH problems [1]. Most people in this MID group live in the community and receive primary (MH) care from local GPs, supported mainly by a mental health nurse practitioner (MHNP). Previous research shows that GP practices often do not recognise the presence of MID in patients [12]. Although GPs acknowledge their role in MH care in general, they only sometimes feel equipped to provide this care [13,14], feeling even less competent providing care to people with a combination of MID and MH problems [5,15,16].

In the Netherlands, people with both MID and MH problems have a high use of primary care services; they receive more consultations and are prescribed more psychotropics than people without ID or with MID alone. This puts a high demand on primary care practices [12].

A recent review confirms GPs’ vital role in providing care to people with MID and MH problems but also indicates that current GP care is often insufficient in terms of underdiagnosis of MH disorders, overmedication, and lack of effective patient follow-up [5]. None of the included publications focused on patients’ perspectives concerning primary MH care for people with MID [5]. Some studies focused on patients’ perspectives on general primary care for people with (M)ID and primary MH care. In these studies, patients with MID indicate that using simple language, sufficient time and the GP’s familiarity with patients with MID, facilitate accessibility and consultations in primary care [17,18]. Patients with MH problems but without MID emphasise the importance of feeling heard, known, and safe at their GP [19]. In light of the above, the combination of MID and MH problems will entail greater or additional needs regarding the GP.

To our knowledge, no studies have explored the perspectives of patients with MID regarding primary MH care. To improve primary MH care in line with the needs of such patients, it is important to stay close to the real-life experiences of patients to whom this care is provided. Therefore, this study aimed to explore patients with MID’s experiences, needs, and suggestions for improvement regarding primary MH care.

## Method

### Setting and sample

For this interview study, we recruited adult patients with MID and MH problems enrolled in GP practices in various Dutch regions between May and October 2022. Our recruitment strategy entailed convenience sampling by sending informational study brochures and flyers to our professional networks within the following organisations: the Radboudumc Academic GP network, national GP and MHNP organisations and advocacy organisations for people with MID. Candidates could apply directly or through their GP or MHNP. Selection criteria for inclusion were: having visited a general practice with MH problems in the previous 12 months, being communicative and mentally capable of participating in an interview, and having a confirmed MID or strongly suspected of having one by their GP or MHNP.

Candidates received easy-to-read study information and both written and verbal informed consent were obtained prior to the interview. If they had a legal representative, their consent was additionally required before participation [20]. In order to create an environment in which the participants felt as free as possible to talk about their own experiences and needs regarding this sensitive subject, interviews were conducted at a location of the participant’s choice, including the option of an online interview, and accompanied by a trusted person when desired. When a trusted person was present, that person did not participate actively in the interview and their personal opinion was not considered in the data analysis. Participants were offered a €15 gift voucher after the interview. The Medical Research Ethics Committee East-Netherland, registration number: 2022-13687, ruled that this study was not subject to the Medical Research Involving Human Subjects Act.

### Data collection

Semi-structured interviews followed an interview guide based on the 11 dimensions of the Person-Centred Primary Care Measures (PCPCM, Supplementary Table 1) [21]. The PCPCM is a patient-reported instrument encompassing 11 primary care aspects considered responsible for primary care effects on population health, equity, quality, and sustainable expenditure. A twelfth dimension, self-determination, was added to the interview guide after expert consultation with the study’s advisory group, which consisted of representatives from patient associations, primary care, ID care, MH care, and addiction care. Self-determination, the freedom to make one’s own choices, whether or not supported by carers, is associated with higher psychological well-being in people with MID and is described in the Convention on the Rights of Persons with Disabilities [22,23].

The interviews were guided around the patient journey in primary care, starting with accessing primary care, followed by assessment, diagnosis, treatment plan including referrals and medication prescriptions and follow-up. A co-researcher (AC) with MID was involved in drafting the interview guide to ensure the clarity of the questions and the adequacy of the sequence of topics for the participants. To tailor the interview to each specific participant, prior to the interview, basic information on the MH care provided was retrieved through the patients and, when there was patient consent, their GP. This information consisted of type of MH problem and whether or not the MHNP was involved, the patient was referred, and medication was prescribed. All interviews were conducted by the same female researcher (KP), an ID physician with broad experience in communicating with people with MID.

### Analysis

The interviews were audio-recorded and transcribed verbatim. The transcripts were analysed following the principles of thematic analysis [24], supported by ATLAS.ti software (version 22.0.11). Two researchers conducted coding independently (KP and MK or MM). Codes were then collated into preliminary themes and repeatedly discussed with the research team (KP, MK, MM, SL, WA, GL) to refine and define the themes. Before the final themes were established, they were discussed and deliberated with the study’s advisory board. The COREQ criteria list for qualitative research was used to guide the analysis and report [25].

## Results

In total, 11 persons with MID (10 females), were interviewed between June 2022 and October 2022, age range 23-63 years. The duration of the interviews was 40 to 70 min. One interview took place online and in three interviews, a trusted person was present. Eight participants received care from an MHNP in the primary care setting (Table 1).

**Table 1.** Characteristics of participants.

Participant	Sex^1^	Age	MH problem^2^	MHNP^3^ involved	Psychotropic(s) prescribed	Referred to MH^2^ services	Trusted person present	Online / face-to-face^4^
1	F	62	Mood problems	X		X		FF
2	F	63	Anxiety	X	X	X	X	FF
3	F	28	Borderline personality Autism Mood problems		X	X		O
4	F	49	Trauma Mood problems	X		X		FF
5	F	26	Anxiety	X		X		FF
6	F	38	Trauma	X	X	X		FF
7	F	50	Depression Panic Trauma	X	X	X		FF
8	F	53	Trauma	X	X	X	X	FF
9	F	61	Mood problems Trauma	X	X	X		FF
10	M	51	Sleep problems		X	X	X	FF
11	F	23	Stress					FF

^1^ Sex: M = male, F = female;.

^2^ MH = mental health problem accordingly to the GP or the participant;.

^3^ MHNP = mental health nurse practitioner;.

^4^ Online/face-to-face**:** O = interview was held online, FF = interview was held face-to-face.

The analysis resulted in four not-mutually exclusive themes. The first, overarching, theme is cumulative vulnerability, relating to the vulnerability – instigated by their MID and reinforced by MH problems – experienced by the participants visiting their GP. Three themes further describe the needs arising from this vulnerability: patient needs regarding the GP, the network and self-determination regarding the MH trajectory (Box 1). Our analysis showed that, self-determination excluded, three levels – cognitive, practical, and emotional – could be identified within these themes. For the sake of readability, GP is stated where it applies to both GP and MHNP. When necessary, GP or MHNP is specified.

Box 1. The perspective of patients with a mild intellectual disability (MID) on primary mental health (MH) care; four themes^1^

*Theme 1: Cumulative vulnerability*

Vulnerability instigated by patients’ MID and reinforced by MH problems.

Barriers and problems experienced:

- Patients themselves recognising and assessing MH problems
- GPs’ accessibility for MH problems
- Information transfer between GP and patients affected by lower cognitive skills and emotions
- Translation of GP’s advice to daily life
- Limited support from a formal and an informal network
- Concerns about being stigmatised or misunderstood by the GP
- Negative past experiences in care and support

Because of this vulnerability, patients have additional needs and expectations regarding the GP, the network, and self-determination regarding their MH trajectory:

*Theme 2: Needs regarding the GP*

- Tailoring communication to the MID
- Securing the GP is easily accessible for MH problems
- Supporting the implementation of given advices, both medical and non-medical
- Overseeing and coordinating all care related to the participant, both medical and non-medical
- Investing in a good patient–GP relationship
- Having knowledge about the patient and his/her context
- Showing initiative to involve the formal and informal network when needed
- Proactively asking questions about possible MH problems

*Theme 3: Needs regarding the network*

- Signalising MH problems
- Encouraging the patient to visit the GP when deemed necessary
- Support with information transfer, organisational tasks, and translation of GP’s advice to daily life
- Providing emotional support

*Theme 4: Self-determination*

Despite their vulnerabilities and additional needs regarding their GP and network, participants expressed the desire for self-determination concerning their MH trajectory1Themes are not-mutually exclusive

### Cumulative vulnerability

Participants experienced a wide range and accumulation of additional barriers and problems occurring in their lives when experiencing MH problems, creating cumulative vulnerability. Compared with GP consultations for somatic problems, most participants experienced extra difficulties visiting their GP for MH problems. These entailed, on a cognitive level, finding it hard to recognise MH problems in the first place and, secondly, to assess whether or not to visit a GP for these problems.

*‘If you have an ear or a stomach ache, you just think these things often happen. But now I wondered if there was something wrong with me. Am I not exaggerating?’ (P1)*

Further, participants encountered communication problems during GP consultations because the GP took insufficient account of the MID. The GP used complex language, had limited time, needed to repeat the given information more frequently and provided general information on MH problems that the participants found difficult to apply to their own specific situation. For example, one participant experienced MH problems after she lost contact with her mother. In this context, the MHNP used a term unclear to her:

*‘Then she said that this was a ‘grieving process’. That wasn’t completely clear to me.’ (P5)*

On both a cognitive and a practical level, participants appeared vulnerable regarding healthcare access and utility of care. For a number of participants, it was not clear that a GP was accessible for MH problems, other than for a specific question about medication.

*‘It feels strange. You think that if you have a stomach ache you can look for help. But if you have other mental problems then you wonder where you can go. You just don’t know.’ (P4)*

After the consultation, it was frequently not possible for participants to follow GP advice. Advice did not sufficiently meet participants’ expectations or was too difficult to implement in their daily lives and not followed at all. One participant, for example, had been advised to write down her thoughts, which she was not able to do:

*‘But at that moment, I also had to write things. But that didn’t really work for me.’ (P5)*

Finally, participants often needed a network offering the practical and emotional support needed to implement GP advice following GP contacts. Frequently, no network was available or participants experienced poor relations with their network. Also, MH problems themselves could be perceived as a barrier to involving network relations: participants indicated feeling uncomfortable talking about their MH in the presence of the network and others, as seen in the following quote, did not want to burden their spouse with their problems.

*‘I can’t bother my husband with this. He has enough problems with me now as it is. Because of this [the MH problems], my relationship isn’t as good as it should be.’ (P9)*

MH problems are often accompanied by strong emotions that, according to the participants, make it even more difficult to open up to their GP and express their symptoms.

*‘Usually, if I’m sad, then I just shut down. And then they [the GP and the MHNP] know that they sometimes have to drag the words out of me. And that I lie a little bit then sometimes. Then I just avoid things and say that there isn’t a problem.’ (P11)*

In addition, participants had concerns about being stigmatised by MH problems or misunderstood by their GP and that, once they were known to have MH problems, somatic problems would be dismissed as psychological.

*‘That they’ll just take pity on you and then you get a ‘P’ on your medical record … so that all of your problems are seen as psychiatric.’ (P1) [Category P in primary care coding stands for ‘known to have MH problems’, note author KP]*

Negative past experiences with GPs could reinforce these concerns but also with MH services and their support network. Finally, participants found it difficult to express dissatisfaction with a consultation or advice or to acknowledge that they did not understand the information given, mainly because of a feeling of shame and fear of disrupting the GP-patient relationship.

### Patient needs regarding the GP

Participants considered it important for the GP and staff to take the MID into account during communication, without the participant having to remind them of their MID. They would like information provided in easy language, repeated several times, and focused on each patient’s specific care situation.

*‘…and if there are any information brochures, they are so general. So not specifically for my problems.’ (P3)*

Participants emphasised the relevance of knowing that the GP is accessible for MH problems and somatic complaints. Easy-to-read information in a leaflet or on the GP website was mentioned as helpful in this respect. In addition, they expect GPs to promptly reply to their needs and actively support the implementation of given medical and non-medical advice.

*‘…just like a daycare programme or something like that. You often had to look for one yourself. And sometimes it’s great that, for example, the GP knows about a programme somewhere. So you don’t have to look for it all by yourself.’ (P3)*

The GP is expected to oversee and coordinate all care, medical and non-medical, related to the patient. In the case of referral to MH services, the GP remains the point of contact for the patient’s MH problem and is expected to regularly and actively inform both MH services and the patient and resolve any barriers to MH care experienced by the patient.

*‘And it is important that the GP questions the organisations. Questions that I can not ask. That she does that for me. Or someone else does it for me because I can’t do it. Especially if I’m having serious mental problems.’ (P4)*

All participants mentioned the importance of a good relationship with the GP, which was considered even more valuable when experiencing MH problems than when experiencing somatic problems. A good relationship, which, according to the participants, includes knowledge about the patient’s past and context, is seen as a prerequisite for opening up during consultations and accepting and following GP advice.

*‘She’s like my confidential advisor … I feel safe with my GP … the attention that someone, the feeling that someone sees you. For the first time in my life, I talked to someone [about traumas in her youth].’ (P4)*

Some participants had a better relationship with their MHNP than with their GP. Aspects considered important in this respect concerned the MHNP having more time to talk, the contact being perceived as less formal, and participants being less worried that somatic problems would be dismissed as psychological.

*‘When I go to my GP, the appointment is very short … but, when I go to S [MHNP], I can really explain the problem. I can talk about things. I need time to talk what I want to say, but that isn’t possible at the GP’s.’ (P8)*

As participants found it difficult to talk about their MH problems, GPs were expected to initiate questions on MH problems, continue to explore the full details of MH problems, and show initiative involving the participant’s network.

*‘Then it’s important to have someone who keeps asking me questions. Also I’m afraid to say or I don’t know if this is something psychological. And that’s really difficult for the GP because they can’t read my mind.’ (P4)*

### Patient needs regarding the network

Participants indicated that they valued the opinions and support of their network – both family members and professional carers. Participants need the network to signal and assess MH problems and encourage contact with the GP when deemed necessary.

*‘And then I kept things to myself for a very long time, like anger. And then my carer said that perhaps I should return to the MHNP.’ (P5)*

In addition, participants found it helpful when someone from the network was present during consultations, as the information provided can be repeated by, and discussed with, that person in the home setting.

*‘She remembers things better. She’s a bit like a second head. So then we talk about it again later.’ (P5)*

Practically, persons from the network can help tailor GP advice to the patient’s daily life.

*‘Then she [the GP] gave my carer some tips. And then she wrote some suggestions on paper, like: what do I do when I feel like this or that? Something like that, those sorts of questions … and my carer developed these further.’ (P6)*

Participants, however, also desired emotional support from their network, both during and after a consultation, especially when feeling vulnerable to being misunderstood by their GP.

*‘Then I felt like no one understood me and I was fed up. And then I contacted a carer … and then I had her explain things for me.’ (P6)*

### Self-determination

Participants emphasised the need for self-determination regarding their MH trajectory. Despite their vulnerabilities and needs regarding their GP and network, they wished to establish their own MH treatment plan and actions in close cooperation with the GP.

*‘She talks about her ideas and I talk about my ideas and together we find a compromise.’ (P7)*

If the GP deems it necessary, the network is allowed to be actively involved, as long as the patient can still make their own decisions, potentially with support from the network if the patient desires that. Participants argued that this may only be overruled if their mental state demands that.

*‘I was really in a bad state so I had to. They had to take action. The GP said ‘Now we’re going to do this’. … My parents were involved in everything. … Now that I feel better and can explain whether or not I’m okay, my parents no longer go with me to the GP’s.’ (P3)*

## Discussion

### Main findings

In this interview study, we aimed to explore patients with MID’s experiences, needs, and suggestions for improvement regarding primary MH care. The results reveal that people with MID feel more vulnerable visiting their GP with MH problems than with somatic problems; this cumulative vulnerability is instigated by their MID and reinforced by MH problems. This translates into various needs and expectations regarding the patient’s GP, family, and professional carers but self-determination remains important.

### Strengths and limitations

To our knowledge, this is the first study to focus on perspectives of patients with MID and MH problems in primary care. To optimise the quality of data collection, interview questions were grounded in the PCPCM structure, evaluated by the study’s advisory board, and simplified and adjusted with the help of a co-researcher with MID. In addition, the interviews were conducted by a researcher who, as an ID physician, has excellent experience in discussing sensitive topics with people with MID. To optimise the quality of data analysis, the research team and advisory board were composed of professionals from diverse backgrounds: patient associations, primary care, ID care, MH care, addiction care, and a research institute. This ensured that various perspectives were included in the data aggregation and theme formation. The discussion among these professionals ensured that personal experiences and related convictions of the research team could only minimally influence the interpretation of the results.

Participants were asked to reflect upon their past experiences with primary MH care – an exercise that might lead to recall bias. We minimised this by including only people who had received primary MH care in the previous 12 months. In addition, selection bias may have occurred for several reasons. First, precise information on participants’ intellectual and adaptive functioning was unavailable. Theoretically, persons with more severe ID or without ID may have been included as participants. However, the expert judgement of the clinical researcher, an ID physician, qualified all participants as persons with MID. Secondly, as most participants were recruited through their GP, people whose MID was not identified by the GP were not invited to participate in this study. Thirdly, only people who were communicative and mentally capable could participate. Finally, we were able to include only one male, despite extra efforts to include more male participants. However, there was a good spread in age and type of MH problems among the 11 participants (Table 1) who were contacted across GP practices throughout the country. These biases may have underexposed specific perspectives.

### Comparison with existing literature

Our findings show some similarities with studies focusing on primary care experiences of people with MID alone and people with MH problems without ID. Like the participants in our study, people with MID in general experience difficulties in contemplating whether or not to visit their GP and in communicating with their GP [17,26]; issues acknowledged by GPs [27–29]. The support of a network, a good relationship with the GP, continuity of care, and autonomy were also deemed important in previous studies [17,27].

In addition, our results overlap with a qualitative study focusing on people in primary care with MH problems in general, where patients also indicated that it is essential to have a good relationship with their GP and for the GP to be familiar with their context and keep up-to-date about their mental wellbeing [19]. In contrast to participants with MID in our study, that study focuses less on practical needs and the role of the network seems less prominent.

### Implications for research and practice

The results of this study provide insight into patients’ expectations and needs and thus offer opportunities to improve the quality of primary MH care for people with MID. Participants expressed the need for the GP and staff to acknowledge their MID. For this, the GP and staff need to recognise a (possible) MID, record this in the patient’s file, and adjust their approach and communication accordingly. To accomplish this, it is relevant to invest in associated training programmes for GPs and staff, to create GP awareness of supporting tools such as screeners for (M)ID, for example SCIL and HASI and to invest in ways to support information exchange [30–32].

GPs were expected to initiate questions on MH problems, as participants found it difficult to talk about their MH problems. Although this can be done during any regular GP consultation, we know from previous research that standardised screening for MH problems may be helpful considering MH problems at an early stage [5]. A periodic (mental) health assessment instrument could be a valuable tool in this regard, and some have already been developed and are available for GPs [16,33,34]. However, further feasibility and (cost-) effectiveness research is necessary.

Participants expected the GP to support them with implementating medical and non-medical advice in their daily lives. GPs should be aware that people with MID may lack practical skills and ensure the involvement of people providing support. Support may come from the patient’s own network, community disability teams, ID care facilities, or, in the Dutch situation, the MHNP or independent client supporters [35]. These supports should be findable and easily accessible for both the GP and the patient, have experience with people with MID, and have a good overview of (care) programmes in the region that are accessible for people with MID.

Some participants found it difficult to coordinate their own care when experiencing MH problems and expected the GP to take on a role as director of their overall longitudinal care, including the organisation of support in daily living situations. However, the question is whether GPs are properly required and equipped to direct this full range of care, especially when the main care focus is non-medical. Collaborative care with the patient, the patient’s network, and other (care) professionals may be more suitable.

Participants expressed the need for a long-term trust relationship with the involved care professionals, and continuity is therefore deemed valuable for this vulnerable patient group. Consequently, the practice organisation should aim at optimal continuity of care provided by the same person [36]. This requires extra time and effort for the GP, which is not always feasible in daily practice. An MHNP or the patient’s own network may be actively involved in supporting and meeting these emotional needs.

Participants desired to maintain self-determination over their MH trajectory in primary care. The ability to make their own decisions contributes to personal well-being in people with ID and/or MH problems [37,38]. To safeguard autonomy in patients with MID, GPs should be aware that this requires additional, patient-tailored, practical and emotional support, taking into account the patient’s cognitive skills. Extra consultation time, accessible information, sufficient alternatives, and someone to talk to are seen as essential for decision-making and should be facilitated in primary MH care [37].

Finally, it would be of additional value to study GPs’ perspectives on primary MH care for people with MID. This may help ensure that suggested implications for practice are feasible in GPs’ daily practice and match these professionals’ needs and capacities.

## Conclusion

The patients’ perspective shows that people with MID, with additional MH problems, feel extra vulnerable in accessing and utilising primary care and desire self-determination over their MH. Their perceived vulnerability requires investment in a good GP–patient relationship and the organisation of additional support to meet their needs. This support is cital for implementing GP advices and coordination of care, whereby patients’ cognitive, practical, and emotional level of functioning is taken into account. This is not only a task for the GP; it also requires collaborative care with the patient, the patient’s network, and other (care) professionals.

## Supplementary Material

Supplemental Material
