# Supplementary material for: Primary mental healthcare for adults with mild intellectual disabilities: Patients’ perspectives
Source: Eur J Gen Pract. 2024 May 17;30(1):2354414. doi: 10.1080/13814788.2024.2354414 (PMC11104687; doi:10.1080/13814788.2024.2354414)
Supplement: Supplemental Material [file IGEN_A_2354414_SM6273.pdf]

Supplementary Table 1<br>*Interview guide for the study:*<br>*Primary mental healthcare for adults with mild intellectual disabilities: patients' perspectives*	
**Standard questions**	**PCPCM[1] dimensions**
What was it like for you to talk to your GP about your mental problems?	Accessibility<br>Relationship
Were you able to talk easily to your GP about your situation?	Comprehensiveness<br>Goal-oriented care<br>Integrating care<br>Family context<br>Community context
Could your GP explain the cause of your problems to you?	Relationship<br>Disease, illness, and prevention management
How did the GP explain how he or she was going to help you?	Disease, illness, and prevention management<br>Shared decision making<br>Relationship<br>Goal-oriented care<br>Family context<br>Community context<br>Advocacy
How did your GP monitor your mental health problems?	Goal-oriented care<br>Continuity of care<br>Advocacy<br>Disease, illness, and prevention management<br>Relationship
What or who helps you to remain mentally healthy?	Disease, illness, and prevention management<br>Advocacy<br>Accessibility
Have you ever talked with your GP or mental health nurse practitioner about your learning problems/(possible) mild intellectual disability?	Accessibility
**Optional questions**	**PCPCM[1] dimensions**
What did you think about being given medicine for your problems?	Relationship<br>Disease, illness, and prevention management
How did you feel about being referred?	Relationship<br>Comprehensiveness<br>Advocacy<br>Coordination<br>Continuity of care
What was it like to be referred to the mental health nurse practitioner?	Relationship<br>Coordination of care<br>Goal-oriented care<br>Comprehensiveness<br>Family context<br>Community context

[1]Based on 11 Person-Centred Primary Care Measures
